# Allelic Variation of *Helicobacter pylori vacA* Gene and Its Association with Gastric Pathologies in Clinical Samples Collected in Jordan

**DOI:** 10.3390/microorganisms13081841

**Published:** 2025-08-07

**Authors:** Mamoon M. Al-Hyassat, Hala I. Al-Daghistani, Lubna F. Abu-Niaaj, Sima Zein, Talal Al-Qaisi

**Affiliations:** 1 Department of Medical Laboratory Sciences, Faculty of Allied Medical Sciences, Al-Ahliyya Amman University, Amman 19328, Jordan; ma.17249@khcc.jo (M.M.A.-H.); h.aldaghistani@ammanu.edu.jo (H.I.A.-D.); 2 Department of Agricultural and Life Sciences, John W. Garland College of Engineering, Science, Technology and Agriculture, Central State University, Wilberforce, OH 45384, USA; 3 Department of Pharmaceutical Biotechnology, Faculty of Allied Medical Sciences, Al-Ahliyya Amman University, Amman 19328, Jordan; s.zein@ammanu.edu.jo; 4 Department of Biomedical Sciences, College of Health Sciences, Abu Dhabi University, Abu Dhabi P.O. Box 59911, United Arab Emirates; talal.alqaisi@adu.ac.ae

**Keywords:** *Helicobacter pylori*, *vacA* gene, allelic variants of *vacA*, vacAm allele, vacAs allele, gastritis, gastric ulcer, gastric malignancy

## Abstract

*Helicobacter pylori* is a well-established causative agent of gastritis, peptic ulcers, gastric adenocarcinoma, and primary gastric lymphoma. It colonizes the human stomach and expresses numerous virulent factors that influence disease progression. Among these factors is the cytotoxin *vacA* gene, which encodes the vacuolating capacity of the cytotoxin and plays a key role in the bacterium’s pathogenic potential. This study investigated the allelic diversity of the *vacA* among *H. pylori* strains infecting patients in Jordan with various gastric conditions and examined potential associations between *vacA s-and m-* genotypes, histopathological and endoscopic findings, and the development of gastric diseases. Gastric biopsies were collected from 106 patients at two hospitals in Jordan who underwent endoscopic examination. The collected biopsies for each patient were subjected to histopathological assessment, urease detection using the Rapid Urease Test (RUT), a diagnostic test for *H. pylori*, and molecular detection of the *vacA* gene and its s and m alleles. The histopathology reports indicated that 83 of 106 patients exhibited gastric disorders, of which 81 samples showed features associated with *H. pylori* infection. The RUT was positive in 76 of 106 with an accuracy of 93.8%. Real-time polymerase chain reaction (RT-PCR) targeting the 16S rRNA gene confirmed the presence of *H. pylori* in 79 of 81 histologically diagnosed cases as infected (97.5%), while the *vacA* gene was detected only in 75 samples (~95%). To explore genetic diversity, PCR-amplified fragments underwent sequence analysis of the *vacA* gene. The m-allele was detected in 58 samples (73%), the s-allele was detected in 45 (57%), while both alleles were not detected in 13% of samples. The predominant genotype combination among Jordanians was *vacA* s2/m2 (50%), significantly linked to mild chronic gastritis, followed by s1/m2 (35%) and s1/m1 (11.8%) which are linked to severe gastric conditions including malignancies. Age-and gender-related differences in *vacA* genotype were observed with less virulent s2m2 and s1m2 genotypes predominating in younger adults specially males, while the more virulent m1 genotypes were found exclusively in females and middle-aged patients. Genomic sequencing revealed extensive diversity within *H. pylori*, likely reflecting its long-standing co-evolution with human hosts in Jordan. This genetic variability plays a key role in modulating virulence and influencing clinical outcomes. Comprehensive characterization of *vacA* genotypic variations through whole-genome sequencing is essential to enhance diagnostic precision, strengthen epidemiological surveillance, and inform targeted therapeutic strategies. While this study highlights the significance of the *vacA m* and *s* alleles, future research is recommended in order to investigate the other *vacA* allelic variations, such as the i, d, and c alleles, to achieve a more comprehensive understanding of *H. pylori* pathogenicity and associated disease severity across different strains. These investigations will be crucial for improving diagnostic accuracy and guiding the development of targeted therapeutic strategies.

## 1. Introduction

*Helicobacter pylori* is a helical, Gram-negative, and microaerophilic bacterium. It possesses four-to six sheathed flagella that enables it to swim through viscous environments. This pathogenic bacterium is a major contributor to a wide range of gastrointestinal diseases, particularly those associated with infection, peptic ulcer, and malignancy [1,2]. Since its dentification in the early 1980s, *H. pylori* has been recognized for its strong affinity for the gastric mucosa, where its persistent colonization plays a key role in the development of chronic gastritis, peptic ulcers, gastric adenocarcinoma, and primary gastric B-cell lymphoma [3]. The development and severity of these conditions are influenced by a complex interplay of host genetic predisposition, environmental factors including the microbiota, smoking and the expressed virulence factors of the infecting strain [4,5,6].

According to the data, advanced-stage gastric cancer (GC) ranks as the fourth leading cause of cancer-related mortality worldwide [7]. It is a heterogeneous malignancy, characterized by diverse molecular and genetic alterations [8]. The prevalence of *H. pylori* infection varies globally, ranging from 30 to 50% in developed countries to over 80% in developing countries [1]. The International Agency for Research on Cancer (IARC), a branch of the World Health Organization (WHO), estimated that half of the global population may be infected with *H. pylori*. In 1994, IARC classified this bacterium as a Group I carcinogen, indicating its established role in human carcinogenesis [9,10,11]. This classification is supported by strong evidence showing that long-term colonization by *H. pylori* is a well-established etiological factor in developing ulcers and gastric malignancies, notably gastric adenocarcinoma and gastric mucosa-associated lymphoid tissue (MALT) lymphoma [10].

Studies suggest that *H. pylori* colonizes the gastric mucosa shortly after birth and may persist throughout life unless eradicated by targeted therapy [12]. The infection often remains clinically silent until pathological changes in the gastric lining become apparent [13]. Following colonization, *H. pylori* can induce acute gastritis through the release of virulence factors. If left untreated, the infection may persist for years, promoting a cascade of histopathological alterations in the gastric mucosa including disruption of intercellular junctions, cell proliferation, and apoptosis. These cellular changes contribute to chronic inflammation and immunomodulation, which ultimately lead to severe disorders and increase the risk of developing gastric malignancy [14,15].

**Figure 1:**
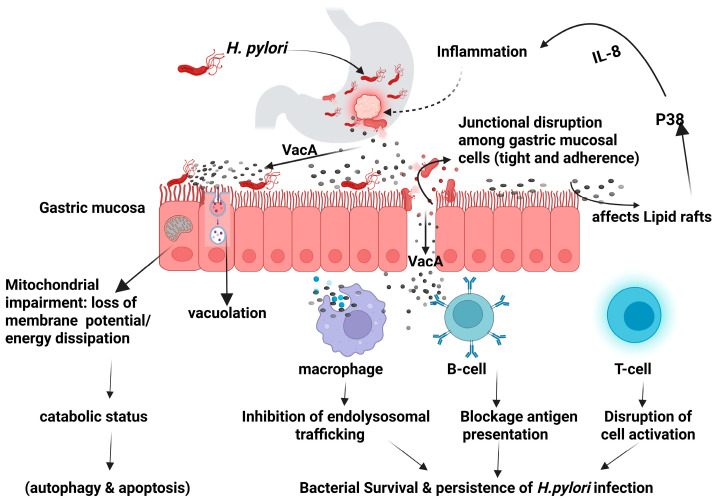
*Helicobacter pylori* VacA mechanism of action. Created in BioRender (https://BioRender.com/0ii4orp).

The virulence factors produced by *H. pylori* can modulate the host intracellular signaling pathways leading to dysregulation of the cellular activities and potentially resulting in neoplastic transformation [16]. The secreted virulence factors can either facilitate the bacterial colonization such as BabA, BabB, SabA, OipA, and HopQ, or contribute to gastric pathogenicity including CagA, VacA, and outer membrane vesicles [14,17,18,19]. Vacuolating cytotoxin A (VacA) is a high molecular multimeric protein present in all strains of *H. pylori.* It is a secreted multifunctional toxin that induces cell vacuolization, causes dysregulation of endolysosomal trafficking, promotes apoptosis, and immunomodulation as illustrated in Figure 1 [14,20].

The *vacA* gene exhibits a high level of diversity, as it consists of three main polymorphic regions; the signal (s) region, the mid (m) region, and the intermediate (i) region. The s- region represents the genotype variation (s1 or s2-region) in the amino terminal and encodes the signal peptide region. The m-region encodes the host cell binding site and represents the genotypic variation (m1 or m2). The intermediate region is polymorphic (i1, i2 or i3) and is located between the s- and m-regions. The combination of different sequences in the three regions plays a major role in capability of vacuolation Combinations of the allelic variations mainly in the s-region (s1 and s2) and m-region (m1 and m2) of the *vacA* gene, are primarily responsible for the severity of the vacuolating effects of various *H. pylori* strains [20,21,22,23]. Despite the contribution of various genotypes of *H. pylori* to the onset of gastric disorders (gastric ulcers, atrophic gastritis, and gastric carcinomas), none of the identified putative virulence genes have proved to be disease specific. Therefore, investigating the relationship between virulence factors and clinical outcomes of *H. pylori* infection is crucial for the early detection of gastric disorders and to enable prompt therapeutic intervention [24]. Clinical studies highlighted that s2m2 was the most common among patients and was primarily associated with gastritis, a milder clinical outcome, while s1m1 genotype and s1m2, were linked to the appearance of peptic ulcer [25]. It was also reported that strains with the genotype s1m1 produce high levels of toxin in vitro, followed by s1m2, while s2m1 strains produce low toxicity and s2m2 strains produce little or no toxin. The combinations with other *vacA* alleles or virulence genes like *cagA* influence the pathogenic potential of *H. pylori* [26]. For example, among s1m2 strains, those carrying the i1 allele are vacuolating, whereas those with the i2 allele are non-vacuolating, which explains the higher risk of developing gastric cancer in individuals infected with strains carrying the *vacAm1* and *s1* alleles [20,25,26,27].

The genome of *H. pylori* exhibits a remarkable degree of diversity, with DNA sequence polymorphism ranging from 2.7% to 8.0% [28]. This diversity results from inter-strain recombination events as well as the species’ clonal nature [29]. An examination of the housekeeping gene sequences (*atpD*, *scoB*, *glnA*, *and recA*) revealed that strains are grouped according to their geographic regions [30]. It was reported that the common strains in Asia (China, Hong Kong, Japan), and Africa, differ from those in the United States, Latin America, and Europe [31,32,33]. Over time, bacterial diversification may be fueled by changes in the environmental conditions within the stomach, especially if colonized by multiple *Helicobacter* strains, which further increases this diversity, particularly in high-prevalence areas [31,34].

Epidemiological studies indicate that the prevalence of *H. pylori* varies significantly based on racial and ethnic groups, gender, and geographical locations. The prevalence rates are highest in developing countries, often exceeding 70%, whereas in developed nations like the United States and parts of Europe, rates have declined to below 30%. The high rate of infection reported in developing countries was attributed to a combination of socioeconomic, genetic, sanitary, and hygiene-related factors, and misuse of antimicrobial agents particularly during early childhood [4,31,35,36]. Among those infected, around 10% may develop peptic ulcer, 1–3% are at risk of gastric adenocarcinoma, and less than 0.1% may develop MALT lymphoma [37]. *H. pylori* Genome Project (HpGP) has provided critical insights into the extensive geographic and phylogenetic diversity of *H. pylori*, shaped by ancient human migrations and historical demographic events. The strains isolated from Jordan, Iran, and Turkey form a distinct Eurasian subpopulation (hspEurasia2), which reflects the Middle East’s historical role as a nexus of human population movement and genetic admixture. This unique genetic assemblage may influence the bacterium’s pathogenic potential and clinical outcomes in the region. These findings emphasize the importance of region-specific genomic sequencing to deepen the understanding of *H. pylori* population structure and evolution, which can inform future diagnostic and therapeutic strategies that are tailored to Middle Eastern populations [38]. Accordingly, this study focuses on the determination of the frequency of the allelic variation for the m- and s-regions of *H. pylori vacA* gene and its association with gastric pathologies in clinical samples collected in Jordan. The findings contribute to advancing our understanding of the evolution of this bacterium and its potential implications for disease pathogenesis in the Jordanian population.

## 2. Materials and Methods

### 2.1. Gastric Mucosal Biopsy Collection

Ethical approval for sample collection was obtained from the Institutional Review Board (IRB) and the hospitals, as detailed in the appropriate section of the manuscript. Gastric biopsies were collected from 106 patients experiencing abdominal and/or gastric pain who underwent endoscopic examination at two hospitals in Jordan, Al-Bashir and Prince Hamzeh hospitals. Socio-demographic and clinical data were collected, and participants signed a consent form prior to biopsy collection via gastric endoscopy. To confirm infection with *H. pylori*, four gastric biopsies were collected from each patient; two of which were fixed in 10% buffered formalin for histopathological examination, one biopsy was used for urease detection via the Rapid Urease Test (RUT), and the fourth biopsy was preserved in 0.5 mL of normal saline and transported to the laboratory within 4 h under appropriate conditions and stored at −20 °C for DNA extraction and molecular analysis.

#### 2.1.1. Rapid Urease Test

This is a rapid and accurate test of *H. pylori* infection based on detection of urease activity. After gastroscopy, the test was performed promptly using the Pyloplus+ Rapid Urease Test (RUT) kit according to its instructions (Lobrie Medical Technologies, Portsmouth, NH, USA). A gastric biopsy from the antrum mucosa was placed into the designated medium which contains urea, and phenol red as pH indicator. Urease produced by *H. pylori* hydrolyzes urea into ammonia, raising the pH and changing its color from yellow to pink, indicating a positive result. A negative result is recorded when the color of medium remains yellow.

#### 2.1.2. Histopathological Examination

Two biopsies fixed in 10% buffered formalin were processed for histological examination following standard protocols. The processed samples were embedded in paraffin and sectioned at 4 µm thickness and stained using the routine hematoxylin and eosin staining procedure for microscopic visualization. The pathological examination was reported based on the Sydney system for histopathological classification. 

### 2.2. Molecular Study

#### 2.2.1. DNA Extraction

DNA extraction Quick-DNA™ Miniprep plus Kit (Zymo research, Tustin, CA, USA) was used for DNA extraction following the manufacturer’s instructions. Briefly, the frozen biopsy was homogenized and mixed thoroughly with 95 µL of water, 95 µL of tissue buffer, and 10 µL of Proteinase K. The mixture was vortexed for 15 s, then incubated at 55 °C for 2 h to allow tissue digestion. Next, 400 µL of genomic binding buffer was added and mixed gently for 15 s. The mixture was then transferred to a Zymo-Spin™ IIC-XLR column placed in a collection tube and centrifuged at 12,000× *g* for 1 min. The flow-through was discarded and a 400 µL of DNA pre-wash buffer was added to the spin column placed in a new collection tube and centrifuged at 12,000× *g* for 1 min. The flow-through was discarded, and the column was then washed by adding 700 µL of g-DNA wash buffer and centrifuged at 12,000× *g* for 1 min. After discarding the flow-through, a second wash was performed by adding 200 µL of g-DNA wash buffer and centrifuged at 12,000× *g* for 1 min. The spin column was transferred to a clean microcentrifuge tube and a 55 µL of DNA elution buffer was added and incubated for 5 min at room temperature. The column then was centrifuged at 12,000× *g* for 1 min to elute the DNA. The DNA concentration was determined using a Nanodrop spectrophotometer (Nabi UV/Vis NANO Spectrophotometer, Daejeon, Republic of Korea). The DNA samples were stored at −20 °C for future analysis.

#### 2.2.2. Primers Preparation

The primers targeting the *16S rRNA* gene, *vacA* gene and its m and s alleles were designed by the authors using the Primer-BLAST (NCBI) to ensure specificity and optimal annealing conditions. Prior to use, primers were suspended by adding nuclease free water and incubated overnight. An aliquot of the stock solution was then diluted in Tris-EDTA (TE) buffer to obtain the desired concentration. The sequences, product sizes, and annealing temperature of the primers used are listed in Table 1.

#### 2.2.3. DNA Amplification of the 16S rRNA and *vacA* Genes by Real-Time PCR

The designated primers were used to amplify the *16S rRNA* and *vacA* genes, yielding fragments of 395 bp and 560 bp, respectively, in reactions conducted on the Applied Biosystems QuantStudio™ 5D Digital PCR System (Thermo Fisher Scientific, Waltham, MA, USA). DNA amplification was performed according to the instructions of the TB Green^®^ Premix Ex Taq™ (Tli RNaseH Plus, Bulk, Waltham, MA, USA) kit (Takara Bio, Cat. #RR820L). A 2 µL DNA template was added to an 18 µL mixture containing 10 µL TB Green Premix Ex Taq II (2X) (Tli RNaseH Plus, Bulk), 0.4 µL of each forward and reverse primers, 0.4 µL ROX Reference Dye (50X), and 6.8 µL sterile purified water. The DNA amplification was performed according to the Shuttle PCR standard protocol. The cycling conditions included an initial denaturation at 95 °C for 30 s, followed by 40 cycles of denaturation at 95 °C for 3 s followed by annealing/extension at 60 °C for 30 s. The parameters for the melt curve stage (last cycle) were 95 °C for 15 s, 60 °C for 60 s, and a final step at 95 °C for 15 s. The bacterial samples were obtained from the National Collection of Type Cultures (NCTC) and the American Type Culture Collection (ATCC). The positive control was *H. pylori* NCTC 11638 while *E. coli* ATCC 25922 served as a negative control.

#### 2.2.4. Allelic Detection of *vacAs* and *vacAm* by Conventional PCR

The detection of *vacAm* and *vacAs* alleles by the conventional PCR. For each PCR reaction, a 50 µL mixture was prepared according to the instructions of the iNtRon PCR Premix kit (Boca Scientific, Dedham, MA, USA). The mixture consisted of a 5 µL DNA template, added to 45 µL of a solution containing 25 µL PCR Master Mix (i-TaqTM), 1 µL of each forward and reverse primers (10 pmol/µL), and 18 µL distilled water. The amplification for each allele began with an initial denaturation at 94 °C for 120 s, followed by 40 cycles of denaturation at 94 °C for 20 s, annealing at 65 °C for 10 s, and extension at 72 °C for 30 s. The melt curve stage (final cycle) extension was at 72 °C for 5 min for *vacAm*, while it was 3 min for the *vacAs* allele. PCR products were visualized by electrophoresis on a 1% agarose gel prepared in 1x Tris Bromate EDTA (TBE) containing SafeRed stain to visualize PCR products. The gel was run for 45 min, and the PCR products band were visualized using photodocumentation (iBright™ 750 Imaging System) (Thermo Fisher Scientific, Waltham, MA, USA). A DNA ladder of 100–1500 bp was used to confirm the size of the PCR product.

### 2.3. Sequencing of PCR Products of vacA Gene

#### 2.3.1. Cleanup Prior to Sequencing

A single-step enzymatic cleanup was performed using ExoSAP-IT™ Express (Thermo Fisher Scientific, Waltham, MA, USA). The reagent comprises exonuclease I and shrimp alkaline phosphatase to remove residual primers and unincorporated nucleotides (dNTPs) that can interfere with downstream sequencing reactions. In a sterile Eppendorf tube, a 2 µL of ExoSAP-IT™ Express reagent was added to 5 µL of PCR product, and the mixture was gently mixed by vortexing and centrifuged for 60 s. The reaction was incubated at 37 °C for 4 min to allow the degradation of the residual primers and unincorporated dNTPs. Termination of the reaction was performed by heating the mixture at 80 °C for 1 min. The purified PCR products were either kept on ice for immediate use or stored at −20 °C until use in sequencing reactions.

#### 2.3.2. DNA Quantification by Invitrogen Qubit 4 Fluorometer

Precise quantification of nucleic acids is critical to ensure optimal DNA input for sequencing. The double-stranded DNA (dsDNA) quantification was performed using the Invitrogen Qubit 4 Fluorometer, which utilizes fluorescence-based assays to provide sensitive and accurate measurements, with working solutions prepared according to the manufacturer’s instructions. To ensure proper reagent volumes, buffer and BigDye^®^ amounts were calculated based on the DNA concentration obtained by Qubit measurements. Two standards, designated as St1 and St2, were prepared by dispensing 190 µL of the working solution into separate tubes, followed by 10 µL of the respective standard. For sample measurements, 195 µL of working solution was mixed with 5 µL of each DNA sample and incubated in the dark at room temperature for 2 min to enhance dye binding. The fluorescence of standards and samples was measured using the Qubit 4 Fluorometer, and DNA concentrations were determined by analyzing the data with the U-Genie application (version 48.1).

#### 2.3.3. Sequencing the Purified DNA

Sequencing reactions were prepared using the BigDye^®^ Terminator v3.1 Cycle Sequencing Kit (Thermo Fisher, Waltham, MA, USA), in accordance with the manufacturer’s protocol. The kit utilizes fluorescently labeled dideoxynucleotides to terminate DNA strand elongation at specific bases during thermal cycling. Briefly, the sequencing reaction was prepared as follows: For the DNA sequencing, a 2 µL of each forward and reverse primers were initially mixed with BigDye^®^ terminator aliquot (4 µL BigDye^®^ + 2 µL Big Dye buffer). Finally, a 2 µL of the purified PCR product (template) was added and the final volume was adjusted based on the amplicon size. The sequenced products were purified using the BigDye^®^ XTerminator™ Purification Kit (Thermo Fisher, Waltham, MA, USA) to remove unincorporated dye terminators and buffer components. The cleaned sequencing plate was loaded into the SeqStudio™ Genetic Analyzer System (Applied Biosystems, Pittsburgh, PA, USA), and sequencing was performed using the designated run module and cleaning dye per the XTerminator™ protocol.

### 2.4. Statistical Analysis

Data analysis was performed using Statistical Product and Service Solutions (SPSS) version 19.0 software version 19.0 (Chicago, IL, USA). Descriptive analysis such as mean and standard deviation (SD) for continuous variables, frequency and percentages for qualitative variables were performed to illustrate the analysis of parameters and mutation characterization for the study participants. The Chi-square test was conducted to evaluate the relationships between the examination tests. The statistical significance was at *p* < 0.05.

## 3. Results

### 3.1. Profiling and Clinical Characterization of Collected Biopsies

Gastric biopsies for 106 patients were included with participants aged 14 to 89 years, representing a broad spectrum from adolescence to older adulthood. With respect to geographic distribution, 83.9% of participants were from central Jordan, 11.3% from the southern region, and 4.8% from northern Jordan. The endoscopic classification revealed that 55 participants (51.9%) had a normal gastric mucosa, 38 (35.8%) had gastritis, 12 (11.3%) had gastric ulcer, and one patient (0.9%) was diagnosed with gastric carcinoma.

### 3.2. Rapid Urease Testing

The urease test showed a color change from yellow to pink in 76 biopsies (71.6%) indicating the presence of *H. pylori* due to urease production. Conversely, 30 samples (28.4%) were negative as no change in the reagent’s color was observed.

### 3.3. Histopathological and Endoscopic Examinations of Gastric Biopsy

The endoscopic evaluation of the 106 patients revealed that 55 patients (51.9%) had gastric mucosa with no visible abnormalities, 38 (35.8%) presented with varying degrees of gastritis, 12 (11.3%) had gastric ulcers, and one patient (0.9%) was diagnosed with gastric carcinoma. On the other hand, the histological examination of biopsies demonstrated that 83 out of 106 gastric cases exhibited evidence of gastric disorders. Among these, 97.8% (81/83) demonstrated features consistent with gastritis in association with *Helicobacter pylori* infection. Six distinct histological patterns were identified based on tissue integrity, degree of inflammation, and cellular abnormalities. The distribution of patients across these categories is summarized in Table 2. The largest category was the 31 biopsies (29.2%) that appeared normal upon endoscopic examination; however, the histological analysis showed that these samples exhibited mild chronic gastritis. The data analysis revealed a significant association between histological and endoscopic findings (*p* = 0.00).

**Figure 2:**
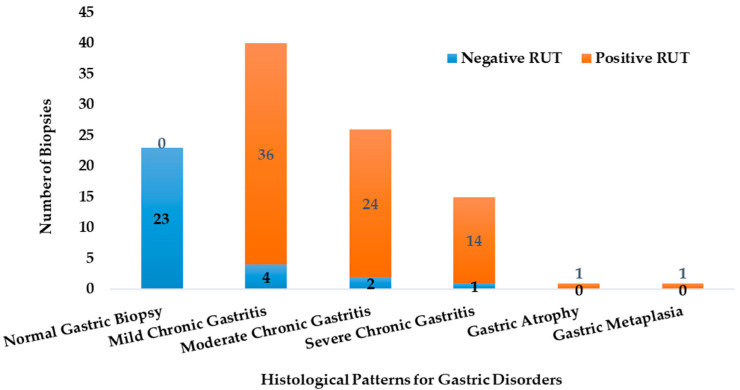
The abundance of the RUT positive and negative biopsies in the different histological categories (n = 106).

The biopsies exhibited varying levels of urease activity, as indicated by the Rapid Urease Test (RUT) results. The RUT was positive by 96% for samples, which were later confirmed by PCR to be infected with *H. pylori.* Figure 2 illustrates how the distribution of RUT-positive and RUT-negative biopsies differs across the various histological profiles.

### 3.4. Molecular Screening of H. pylori

#### 3.4.1. Diagnosis of *H. pylori* Using 16Sr RNA, and *vacA* Genes

**Figure 3:**
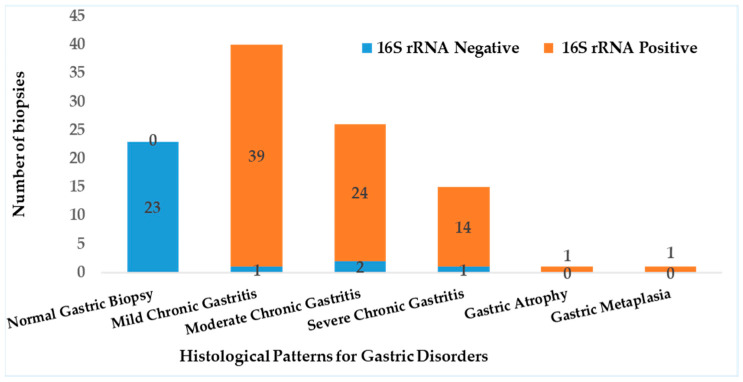
Prevalence of *H. pylori* detected by RT-PCR and it correlation to histopathology.

Detection of *H. pylori* was performed using real-time quantitative PCR (RT-qPCR) targeting the 16S rRNA gene (Figure S1). The assay revealed that 79 out of the 81 cases with gastritis (97.5%) were positive for *H. pylori*. RT-PCR results showed a significant correlation with histopathological findings (*p* < 0.05), and biopsies with normal histology tested negative for *H. pylori* (Figure 3). Out of the 79 samples, only 75 (~95%) harbored *vacA* gene, revealing a statistically significant association with *H. pylori* infection (*p* < 0001).

#### 3.4.2. Allelic Variants Detection of *vacA* by Conventional PCR

**Figure 4:**
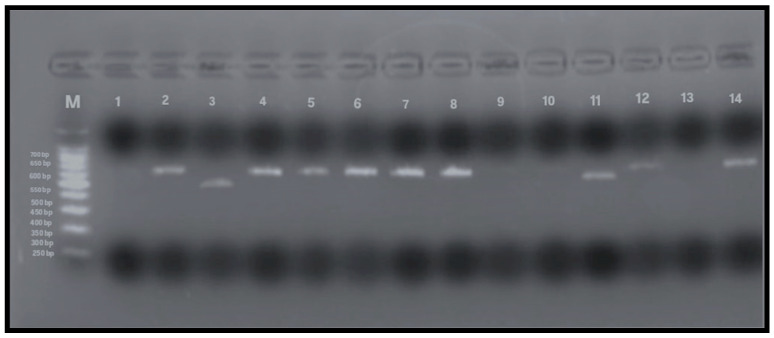
PCR amplification of *vacA* m1 and m2 alleles (567 and 642 bp, respectively). Lane M: 100 bp DNA ladder; Lane 1: negative control; Lane 2: positive control; Lanes 4–8, 12, 14: *vacA* m2-positive samples (642 bp); Lanes 3, 9–11: *vacA* m1-positive samples (567 bp); Lane 13: negative for *vacAm* alleles.

**Figure 5:**
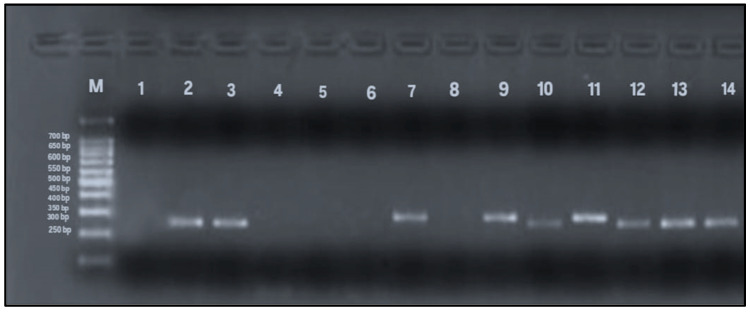
PCR amplification of *vacA* s1 and s2 alleles (259 and 286 bp). Lane M: 100 bp DNA ladder; Lane 1: negative control; Lane 2: positive control; Lanes 3, 10, 12–14: *vacA* s1-positive samples (259 bp); Lanes 7, 9, 11: *vacA* s2-positive samples (286 bp); Lanes 4–6, 8: negative for *vacAs* alleles.

To detect allelic variants (s and m) of the *vacA* gene, PCR analysis using gel electrophoresis was performed on all 79 samples that tested positive for the *Helicobacter pylori* 16S rRNA gene. The expected PCR product sizes were 570 bp and 642 bp for the *vacA* m1 and m2 alleles, respectively, and 259 bp and 286 bp for the s1 and s2 alleles. The presence of these specific bands indicated successful amplification and identification of *vacA* alleles (Figure 4 and Figure 5). Among the 79 samples, the m-region allele was detected in 58 samples (73%), and the s-region allele in 45 samples (57%), while both alleles were undetectable in 13% of the samples. Interestingly, four samples that were negative for *vacA* by RT-qPCR exhibited detectable m- and s-allele bands by conventional PCR and were therefore included in the final calculations (n = 79). 

**Figure 6:**
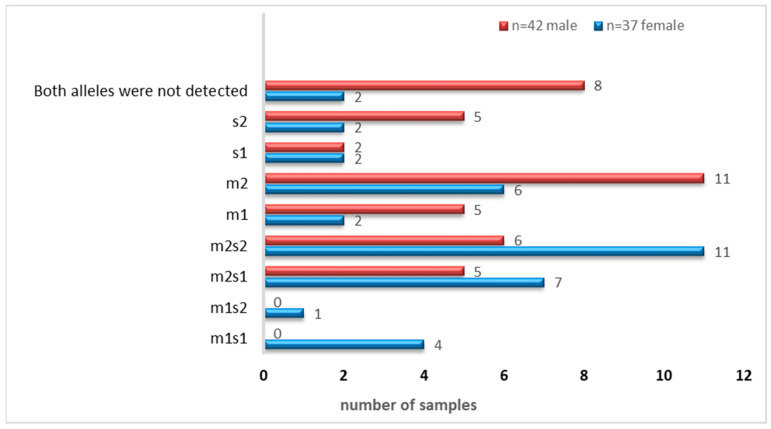
Distribution of *vacA* m-and s-alleles as combinations or individually.

As shown in Table 3, the vm2 and s1 allelic variants were the most frequently identified variants among the analyzed samples. The abundance of the allelic genotypes of the m-and s-regions of *vacA* (combined and individually) is shown in Figure 6.

**Figure 7:**
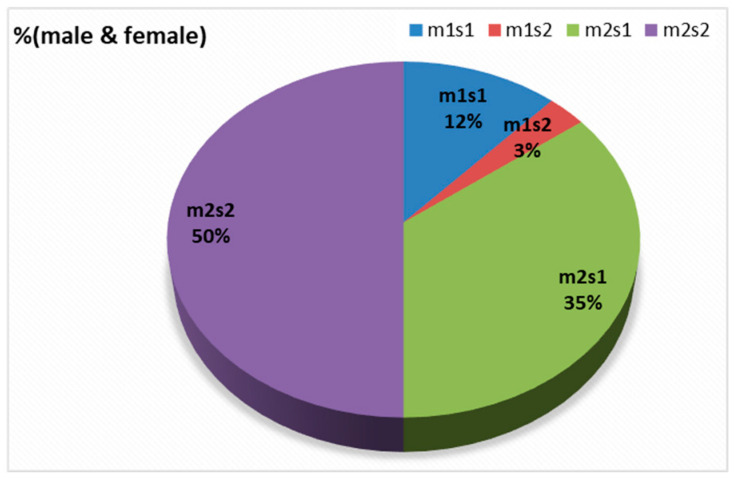
The distribution of the four combinations of m- and s-alleles in tested samples.

**Figure 8:**
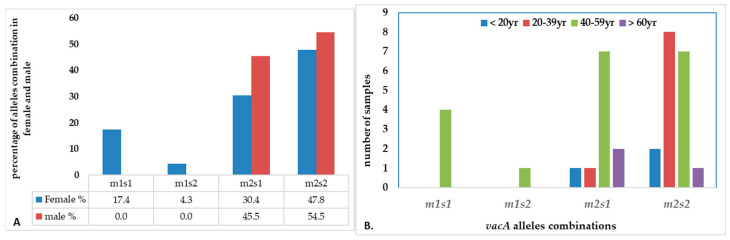
The distribution of combination of *vacA* alleles in (**A**) female vs. male; (**B**) age groups.

**Figure 9:**
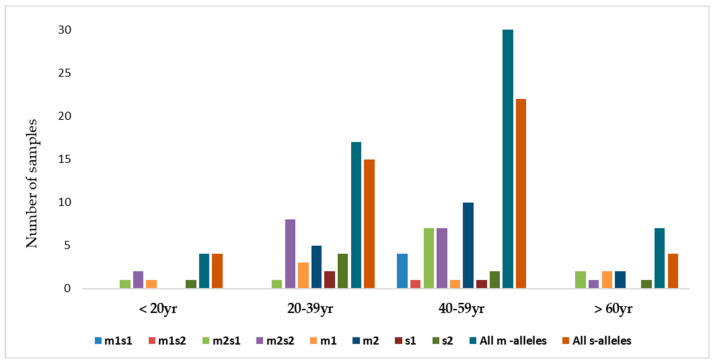
The distribution of combinations of *vacA* allelic variants in different age groups of patients.

There were four combinations of *m* and *s-* genotypes that were detected in 34 out of 79 samples confirmed to be *H. pylori*-positive (~43%) (Figure 7). Such groups showed a variable prevalence in female and male groups, as well as in different age groups. The distribution of the combined genotypes differed by sex, with females (n = 23) and males (n = 11) showing distinct patterns. The s2m2 combination was the most prevalent (50%), followed by s1m2 (35%), while s1m1 and s2m1 are less frequent, and was detected only in female samples (Figure 8A). However, these two combinations (m1s1 and m1s2) are the only allelic genotypes present in younger age groups (<40 yr) as shown in (Figure 8B). The distribution of the allelic variants, as a combination or individual, in different age groups is illustrated in Figure 9.

The correlation of *vacA* variants (m and s) with the endoscopic findings and histological categories was illustrated (Table 4). A significant association between histopathology results and *vacA m and s* variants was detected (*p* < 0.001).

**Figure 10:**
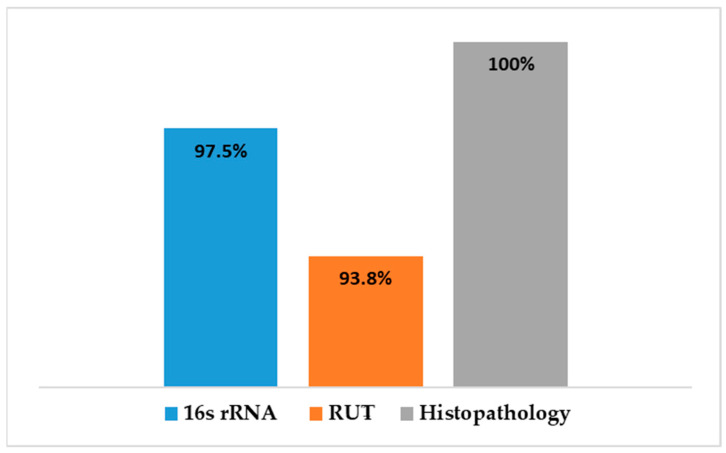
A comparison of the diagnostic methods for *H. pylori* infected gastric tissues.

A comparison was made between the different diagnostic tests used to detect *H. pylori* in gastric tissue is shown in Figure 10. Histopathological examination revealed gastric abnormalities in all 106 patients, with 83 showing gastritis and 81 exhibiting features associated with *H. pylori* infection. PCR targeting the 16S rRNA gene confirmed *H. pylori* presence in 79 of the 81 samples tested (97.5%)**,** while the rapid urease test (RUT) was positive in 76 of 81 cases (93.8%). 

#### 3.4.3. Diversity of *H. pylori* Strains by Sanger Sequencing

**Figure 11:**
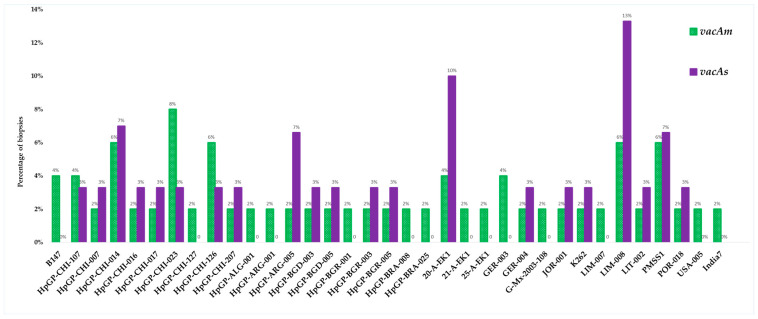
Distribution of *H. pylori* strains identified by sequencing *vacA* allelic variants (m and s).

The purified PCR products were subjected to Sanger sequencing at Biotrust Laboratories (Irbid, Jordan). A comprehensive sequencing of 50 *vacAm* positive samples identified 35 genetically distinct strains with varying pathologic potential, while the sequencing of 30 *vacAs* positive samples identified 21 unique strains (Figure 11).

## 4. Discussion

Infection with *H. pylori* can lead to chronic gastritis and several gastroduodenal diseases, such as peptic ulcers and gastric cancer including mucosa-associated lymphoid tissue (MALT) lymphoma. The infection is often acquired in early life and persists throughout life if untreated. The pathogenicity of *H. pylori* and the clinical outcomes are influenced by a complex interplay between the bacterial genome and the expressed virulence factors, host genetics, and environmental factors [14,39].

One of the most critical virulence factors is the vacuolating cytotoxin A (VacA), which contributes significantly to disease progression in individuals infected with *H. pylori* [21,40]. The *vacA* is among those genes known to modulate host immune response and impact the severity of the bacterial infection. Given its potential as both a diagnostic and prognostic biomarker, this study aimed to detect the allelic variants of the *vacA* gene in clinical samples confirmed to be *H. pylori*-positive (n = 79), and to determine whether these variants are associated with different gastric pathologies in infected patients in Jordan. Gastric biopsies were collected from 106 patients experiencing gastric discomfort or abnormalities. The specimens were subjected to three diagnostic workflows: the rapid urease test (RUT), histopathological assessment, and molecular analysis targeting the 16S rRNA gene. The histopathology reports indicated that 83 of 106 patients exhibited gastric disorders, of which 81 samples showed features associated with *H. pylori* infection. Real-time qPCR of the *H. pylori* 16S rRNA performed on all the clinical samples collected (n = 106), and results showed that 79 of the 81 histologically infected cases were positive (97.5%). The RUT was positive in 76 of 81 cases indicating a diagnostic accuracy of 93.8%. The lower detection by the real-time assay is most likely due to insufficient quality or quantity of the sample DNA. There were no false-positive results obtained either by RUT or 16S rRNA PCR in biopsies deemed uninfected by histopathology, demonstrating a strong concordance among the three diagnostic approaches for this bacterium. Although the missing number of the infected samples is small, relying solely on RUT could adversely affect the proper timely treatment. Therefore, these findings suggest that RUT is the least sensitive method for detecting *H. pylori*, while molecular and histopathology testing are more reliable. The histopathological examination detected gastric abnormalities in all infected biopsies, highlighting its dual value for the assessment of the gastric tissue damage as well as for the bacterial detection. Although these diagnostic methods are considered invasive, though, such findings reinforce the importance of combining molecular and histological approaches for accurate diagnosis of *H. pylori.* As the findings demonstrated, the endoscopic examination revealed a wide spectrum of gastric abnormalities potentially linked to *H. pylori* infection. The conditions range from normal-appearing mucosa to severe abnormalities such as gastritis, ulcers, and carcinoma. Such clinical variation poses a challenge for detecting *H. pylori* during early stages of the infection. However, the histopathological assessment proved more sensitive in detecting underlying gastric damage, identifying microscopic evidence of gastritis in the infected biopsies. A strong correlation was observed between endoscopic abnormalities and histological findings emphasizing the complementary value of these diagnostic modalities in assessing *H. pylori*-related disease. These findings highlight the significant contribution of *H. pylori* to the development of chronic gastritis and the significant statistical association (*p* < 0.05) between endoscopic and histological observations reinforces the diagnostic reliability of their combined use as demonstrated in previous studies, as also demonstrated in studies [41,42]. In Jordan, a study reported *H. pylori* in 78.3% of samples, a prevalence close to the seropositivity (88%) reported among Jordanian populations underscoring the high endemicity of *H. pylori* infection in the country [43]. Transmission of this pathogen primarily occurs via oral–oral or fecal–oral routes, often in early childhood, and the infection frequently persisting asymptomatically into adulthood in the absence of targeted antimicrobial treatment. The elevated prevalence may, in part, reflect limited public awareness regarding transmission routes and preventive health practices [36,44].

The clinical samples confirmed to be *H. pylori* positive based on the 16S rRNA were subsequently tested for the presence of *vacA* by RT-qPCR. The assay detected the gene in most infected tissues but was absent from histologically normal biopsies, reinforcing its association with the development of pathological conditions and its potential as a molecular marker of mucosal damage. This finding aligns with previous reports demonstrating the absence of *vacA* gene in normal gastric tissues, further emphasizing its specificity to pathological conditions and mucosal abnormalities. The *vacA* gene was not universally detected in all *H. pylori* strains, with some studies reported its presence in approximately 90.36% of *H. pylori* strains while its allelic variants were linked to varying levels of cytotoxic activity and disease severity [45,46]. Strains isolated from patients with peptic ulcers exhibit significantly higher cytotoxin activity than those with gastritis alone The detection of *vacA* in 48% of patients with only mild gastritis suggests its expression occurs during early infection [47,48,49]. This supports its potential role as a biomarker for early *H. pylori* colonization and gastric mucosal alterations, rather than limiting it to advanced disease stages.

In this study, the *vacA* gene was detected in 75 of the 79 samples tested positive to *H. pylori* 16S rRNA (95%). Interestingly, the four samples where the *vacA* gene was not detected, PCR still amplified either the m-allele or both the m- and s-alleles. The failure of the RT-qPCR to detect the *vacA* gene was most likely attributed to gene mutations, primer mismatch with specific strain variants, or suboptimal sample quality. This detection rate aligns with studies from regions with similar environmental and socioeconomic conditions where *H. pylori* prevalence was reported at 79% and 81%, respectively [50]. These results highlight the diagnostic value of molecular methods and underscore the persistent burden of *H. pylori* infection in the region. Allelic prevalence varied but was most frequently observed in mild to moderate gastritis. The *vacAm* was detected in 73% of infected samples (58 out of 79), while the *vacAs* was detected in 57% of infected samples (45 out of 79). The findings showed four allelic combinations with varying prevalence across 34 samples (23 female, 11 male). Our results reveal gender- and age-related variation in the distribution of *vacA* genotypes. The distribution of the allelic combination showed differences between males and females. Aligning with the higher total counts of m2 and s2, the estimated distribution of *vacA* allelic combinations combined in female and male (n = 34) revealed that s2/m2 and s1/m2 genotypes were the most prevalent, representing approximately 50% and 35%, respectively. The detection of less virulent allelic combinations of s2/m2 and s1/m2 was at a higher rate among males, while the virulent combinations were absent in this group but exclusively detected in females. In contrast, the more virulent m1/s1 genotype appeared exclusively in females with a prevalence of 12% among the combination groups, while m1/s2 genotype represents only 3% of the four combinations. The complete absence of the both virulent combination of genotypes (m1s1 and m1s2) in male patients suggests a potential gender-based distribution of the combined alleles causing a difference in strain acquisition and host responses. Such finding aligns with some studies which indicated increased prevalence of such combinations among males, possibly due to genetic factors, yet none demonstrated a statistically significant association between gender and *H. pylori* infection risk [51]. Further studies are needed to confirm any significant association between gender and *H. pylori* infection risk.

Our findings showed the high prevalence of *vacA* and its allelic variants significantly influence the cytotoxicity and clinical severity. This suggests a predominance of less virulent *vacA* combinations, which correlates with the higher frequency of non-ulcerative diagnoses such as normal-appearing mucosa and gastritis. The absence of any *vacA* alleles in carcinoma cases limits the ability to withdraw conclusions regarding the association for malignancy association. These patterns may inform the local epidemiology and help predict potential clinical outcomes based on genotype distribution.

These findings align with several studies studying the prevalence of virulence factors of *H. pylori*. A Moroccan study that detected *vacA s2m2* was detected in 47.1% of samples [4]. Despite the high prevalence of *Helicobacter pylori* infection in Jordan, the incidence of gastric cancers accounts for only 2.7% of all newly diagnosed cancer cases [52]. This discrepancy is likely attributable to the bacterial strain characteristics and the type and expression levels of associated virulence factors. The combination of *vacA* allelic variants plays a role in *H. pylori* pathogenicity and the potential development of malignancy. The convergence of our results with findings from diverse geographic settings underscores the robustness and potential clinical utility of *vacA* genotyping as a predictive biomarker for disease risk stratification in *H. pylori*-associated gastric pathology. Studies on populations from the Middle East, Latin America, and Africa had shown that *H. pylori* strains carrying the *vacA* s1 or m1 alleles confer a higher risk for gastric carcinoma and peptic ulcer disease than those with s2 or m2 variants [10,50,52,53,54,55,56]. Data analysis of numerous studies in numerous countries including United States and Japan, revealed a predominance of *vacA s1m1*, the most virulent genotypes in all regions studied, while s2m2 produce fewer active forms of the toxin and are considered less virulent [4]. Such finding is closely aligned with a study in Saudi Arabia, where normal gastric mucosa was observed in 55.6% of cases, followed by gastritis and ulcerative lesions [57]. Similar patterns were also reported in northern Iran which reflected a significant association between histopathology and both *vacA* alleles (*p* = 0.0007 and 0.001, respectively), underscoring the clinical relevance of these genetic variants not only to molecular pathogenicity but also to clinically observable gastric mucosal changes [58]. No significant association was reported between *vacA* genotypes and gastric mucosal pathology [59]. However, the prevalence of *vacAm* falls within a previously reported prevalence range of 24% to 84% [60].

Studies performed in Egypt showed that the *pylori* genotypic combination of *vacA s2m2/iceA1* and *vacAs1m1/cagA* were the most common and predominantly associated with gastritis, while *vacA s1/cagA/iceA1* was more frequently linked to peptic ulcer disease [61,62]. Another study performed on Egyptian patients reported the prevalence of *vacA s1* (42.9%) and *m1* (14.3%) alleles in isolates of *H. pylori* [60]. In contrast, Indian data reported higher rates of s1m2 and s1m1 [63]. These regional differences in *vacA* allele prevalence and their association with disease outcomes highlight the importance of geographic and genetic factors in shaping the clinical manifestations of *H. pylori* infection.

This study reports that the weak cytotoxic genotypes, s2m2 and s1m2, are dominant in Jordan. Studies suggested that the relatively low incidence of gastric cancer in Middle East populations despite the high prevalence of *H. pylori* infection, may be attributed to the predominance of weakly cytotoxic genotypes, particularly *vacAm2s2* [25,64]. These regional differences are likely influenced by various factors including host genetics, bacterial evolution, environmental exposures and transmission dynamics. Our results further reveal an age-related variation in the distribution of *vacA* genotypes. The less virulent genotypes were also more frequently observed in younger adults, suggesting a link between lower pathogenic potential and early stage of infection. In contrast, the more virulent genotypes (m1s1 and m1s2) appeared in middle-aged individuals (40–59 years). The predominance of the less virulent s2m2 and s1m2 genotypes in the Jordanian population explains the relatively mild histopathological findings observed in this study, with mild gastritis as the most common in gastric biopsies. The current study further establishes a more pronounced association between the clinical condition of patients and the *vacA* genotype of the infecting strains. A high degree of nucleotide variation was observed in the *vacA* s and m regions, reflecting substantial genetic heterogeneity of *H. pylori* strains circulating in Jordan. 

This finding aligns with previous studies in Jordan and other regions Reporting the genetic variation in *H. pylori* strains infecting patients at different ages [19,20,36,64,65]. Overall, these genetic differences influence pathogenesis and modulate immune response profiles, thereby guiding advances in diagnostics and therapeutic strategies. Pérez-Pérez et al. reported that IgG antibody levels were significantly higher in patients infected with s1/m1 strains compared to those with s2/m2 or s1/m2 genotypes, underscoring the diagnostic utility of allele-specific immune profiling in *H. pylori* infection [65]. In addition, serum biomarkers, particularly specific anti-inflammatory agents, have been shown to aid in evaluating host immune responses to *H. pylori* infection [66]. Further research has assessed the effectiveness of numerous natural and synthetic compounds targeting *H. pylori* proteins as potential therapeutic agents [67,68]. Overall, allele profiling supports the development of non-invasive diagnostics, vaccines, and targeted antimicrobial therapies against *H. pylori*.

The sequencing analysis in this study reflected a highly diverse sequence of the allelic variants of *vacA (m* and *s)* in distinct strains of *H. pylori* which most likely correlated to the varying pathogenic potential of the bacteria and the histological outcomes. The sequence dataset targeting the *vacAm* identified 35 possible strains (from 50 samples) harboring this allele, while the sequence dataset targeting *vacAs* identified 21 strains (from 30 samples). The strains reflect a broad geographic distribution across the Middle East, South and North America, Asia, Europe, and North Africa. The broader *vacAm* dataset also highlights extensive allelic diversity and geographic distribution, indicating varied virulence potential across strains. Most of the identified strains have been highlighted in the *Helicobacter pylori* Genome Project (*hpGP*). The *hpGP* was launched with the ambitious goal of sequencing 1000 *H. pylori* strains collected from diverse global populations that exhibit varying risks for gastric cancer. This large-scale effort aimed to provide a comprehensive genetic landscape of *H. pylori* by including strains from regions with both high and low gastric cancer incidence. By analyzing these genomes, researchers sought to uncover critical insights into the long-term outcomes of *H. pylori* infection and elucidate genetic associations between persistent colonization and progression through the gastric carcinogenesis cascade [38,69,70]. The project represents a pivotal step in understanding how specific bacterial genotypes may influence host–pathogen interactions, disease severity, and cancer risk across different human populations. Furthermore, it supports ongoing efforts to develop precision vaccines against *H. pylori* tailored to specific populations [70].

Despite the observed correlation between *vacA* allelic variants and clinical outcomes, there are some limitations to this study. The findings may not be applicable to the broader Jordanian population mainly the DNA was extracted directly from gastric biopsies without isolating *H. pylori* strains. It may have also introduced mixed infections, potentially affecting the specificity of genotype-strain and the accuracy of qPCR detection for *vacA.* In addition, the use of gene fragment sequencing without whole-genome analysis restricts a deeper understanding of strain diversity.

In conclusion, although *H. pylori* infection was highly prevalent among Jordanian patients with gastric disorders, the predominance of low-virulence genotypes, particularly *vacA* s2m2, may contribute to the lower rates of severe gastric disease and malignancy. The significant association between the *vacA* s2m2 genotype and mild gastritis highlights the potential of *vacA* genotyping as a reliable molecular marker for assessing *H. pylori* strain virulence and determining the potential clinical risk of the outcomes. While these findings are promising, further whole-genome sequencing of the infecting strain of *H. pylori* and detailed molecular analyses are warranted to fully characterize the genetic diversity of circulating *H. pylori* strains in Jordan.

## Figures and Tables

**Table 1:** Primers sequences used and their products size.

Gene or Allelic Variant	Primer	Primer Sequence (5′-3′)	Product Size (bp)
*vacA*	Forward (F)	AAGTGTGGGGGAATACAC	177
	Reverse (R)	CTAGCGTCAAAATAATTCCAAGG	177
*vacAs*	Forward (F)	ATGGAAATACAACAAACACAC	259–286
	Reverse (R)	CTGCTTGAATGCGCCAAAC	259–286
*vacAm*	Forward (F)	CAATCTGTCCAATCAAGCGAG	567–642
	Reverse (R)	GCGTCAAAATAATTCCAAGG	567–642
*16S rRNA*	Forward (F)	ATTTGCGATTACTAGCGATT	205
	Reverse (R)	AGGATACTGCCTCCGTAAG	205

**Table 2:** Correlation between the histology and endoscopy findings of gastric biopsies.

Findings of the Endoscopy Examination						
Histological Diagnosis	Normal Mucosa	Gastritis	Peptic Ulcer	Gastric Carcinoma	Biopsies (n)	Total %
Normal gastric mucosa	23	0	0	0	23	
	21.7%	0.0%	0.0%	0.0%		21.7%
Mild chronic gastritis	31	9	0	0	40	
	29.2%	8.5%	0.0%	0.0%		37.7%
Moderate chronic gastritis	0	24	2	0	26	
	0.0%	22.6%	1.9%	0.0%		24.5%
Severe chronic gastritis	1	4	10	0	15	
	0.9%	3.8%	9.4%	0.0%		14.1%
Gastric atrophy	0	1	0	0	1	
	0.0%	0.9%	0.0%	0.0%		0.9%
Gastric metaplasia	0	0	0	1	1	
	0.0%	0.0%	0.0%	0.9%		0.9%
**Number of biopsies**	**55**	**38**	**12**	**1**	**106**	
**Percentage of biopsies**	**51.9%**	**35.8%**	**11.3%**	**0.9%**		**~100%**

Bold as they are the total number (not for each categories).

**Table 3:** Allelic detection in the RT-qPCR negative for *vacA* samples (n = 4).

Gender	Count	Age (y)	PCR Detection of *vacA* Alleles
Male	3	39, 58, 58	s2m2, s1m2, s1m1
Female	1	27	m2

**Table 4:** The correlation between the allelic variants *vacAm1*, *m2*, *s1*, *s2*, endoscopic results, and the histological examination of biopsies.

Diagnostic Category	Subcategory	*vacAm1* (n, %)	*vacAm2* (n, %)	*vacAs1* (n, %)	*vacAs2* (n, %)
Endoscopic Findings	Normal gastric mucosa	6 (50.0%)	16 (34.04%)	8 (42.1%)	7 (28.0%)
	Gastritis	4 (33.3%)	24 (51.06%)	9 (47.4%)	15 (60.0%)
	Gastric ulcer	2 (16.7%)	7 (14.9%)	2 (10.5%)	3 (12.0%)
	Gastric carcinoma	0 (0.0%)	0 (0.0%)	0 (0.0%)	0 (0.0%)
Allele positive samples		n = 12	n = 47	n = 19	n = 25
		n = 59 (*vacAm* positive)		n = 44 (*vacAs* positive)	
	*p*-value (* significant)	<0.001 *	<0.001 *	0.021	0.021
Histopathological Findings	Normal gastric mucosa	0 (0.0%)	0 (0.0%)	0 (0.0%)	0 (0.0%)
	Mild gastritis	7 (6.6%)	21 (19.8%)	10 (9.4%)	12 (11.3%)
	Moderate gastritis	2 (1.9%)	15 (14.2%)	6 (5.7%)	7 (6.6%)
	Severe gastritis	3 (2.8%)	11 (10.4%)	3 (2.8%)	6 (5.7%)
	Gastric atrophy	0 (0.0%)	0 (0.0%)	0 (0.0%)	0 (0.0%)
	Gastric metaplasia	0 (0.0%)	0 (0.0%)	0 (0.0%)	0 (0.0%)
allele positive (total)		n = 12	n = 47	n = 19	n = 25
		n = 59 (*vacAm* positive)		n = 44 (*vacAs* positive)	
	*p*-value (* significant)	<0.001 *	<0.001 *	<0.001 *	<0.001 *

## Data Availability

The datasets presented in this article are not readily available because the data involve human gastric biopsy specimens and are subject to ethical and confidentiality restrictions. Requests to access the datasets should be directed to the corresponding author.

## Supplementary Materials

The following supporting information can be downloaded at: https://www.mdpi.com/article/10.3390/microorganisms13081841/s1, Figure S1: Amplification plots of *16SrRNA* (a) and *vacA* gene (b); 1 represents *H. pylori* NCTC 11638 and the rest of curves represent positive samples.
