# Supplementary material for: Allelic Variation of Helicobacter pylori vacA Gene and Its Association with Gastric Pathologies in Clinical Samples Collected in Jordan
Source: Microorganisms. 2025 Aug 7;13(8):1841. doi: 10.3390/microorganisms13081841 (PMC12388764; doi:10.3390/microorganisms13081841)
Supplement: Supplementary file 1 [file microorganisms-13-01841-s001.zip › microorganisms-3697065-supplementary.pdf]

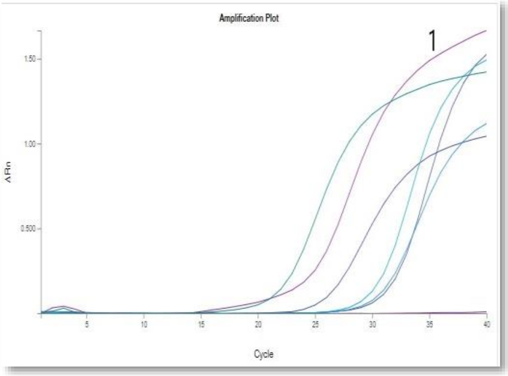

(a) *16SrRNA* gene

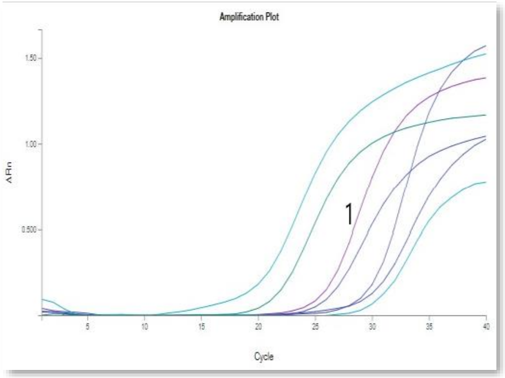

(b) *vacA* gene

Figure S1. Amplification plot of *16SrRNA* (a) and *vacA* gene (b); 1 represents *H. pylori* NCTC 11638 and the rest of curves represent positive samples.
